# Serum proteomic changes related to residual impairment in remittent depression are associated with immune and inflammatory processes

**DOI:** 10.1038/s41598-024-75983-0

**Published:** 2024-10-18

**Authors:** Seungyeon Lee, Sora Mun, Eun-Jeong Joo, Yeeun Yun, Hee-Gyoo Kang, Jiyeong Lee

**Affiliations:** 1 Department of Senior Healthcare, Graduate School, Eulji University, Uijeongbu, 11759 Republic of Korea; 2 Department of Biomedical Laboratory Science, College of Health Sciences, Eulji University, Seongnam, 13135 Republic of Korea; 3 Department of Neuropsychiatry, School of Medicine, Eulji University, Daejeon, 35233 Republic of Korea; 4 Department of Psychiatry, Uijeongbu Eulji Medical Center, Eulji University, Uijeongbu, 11759 Republic of Korea; 5 Department of Biomedical Laboratory Science, Graduate School, Eulji University, Uijeongbu, 11759 Republic of Korea; 6 Department of Biomedical Laboratory Science, College of Health Science, Eulji University, Uijeongbu, 11759 Republic of Korea

**Keywords:** Major depressive disorder, Residual impairment, Remission, Inflammation, Proteomics, Biomarkers, Diseases, Proteomics, Blood proteins

## Abstract

In patients with major depressive disorder, various functional areas are impaired, negatively impacting the quality of life. Remission can restore pre-depression functions; however, some patients may still have residual impairments. Distinguishing between near-normal recovery and residual impairment helps identify those at a high risk of relapse risk and helps tailor treatment. Accordingly, we aimed to discover and validate biomarkers that distinguish between near-normal recovery and residual impairment in remission states through serum proteome analysis. Pooled serum and individual serum samples from three groups (depression status, remission status with residual impairment, and remission status with normal recovery) were analyzed using liquid chromatography-tandem mass spectrometry. The combination of four proteins—antithrombin-III, serum amyloid A4 protein, C1q subcomponent subunit B, and serum amyloid P-component—was selected as a candidate biomarker. The trend of protein changes suggests complement C1q subcomponent subunit B and serum amyloid P-component as potential biomarkers for distinguishing remission from residual impairment. Changes in complement C1q subcomponent subunit B and serum amyloid P-component suggest that the complement system and inflammation-related immune mechanisms are associated with residual impairment in remittent major depressive disorder.

## Introduction

Major depressive disorder (MDD) is a prevalent psychiatric condition characterized by symptoms such as depressed mood, anhedonia, appetite changes, and suicidal thoughts. These symptoms impair psychosocial, academic, and occupational functioning^1^. MDD is widespread globally and ranks among the leading causes of disability ^2–4^. Several hypotheses have been proposed to explain its etiology, including hypothalamic–pituitary–adrenal axis dysfunction, inflammation, and genetic and epigenetic abnormalities. However, the pathophysiological mechanisms of MDD remain to be elucidated ^2,5^. This incomplete understanding, along with the absence of definitive biomarkers, hinders the development of effective diagnostic and therapeutic strategies for MDD.

The primary treatment strategy for MDD is antidepressant therapy^6^, with complete remission as the therapeutic goal^7,8^. Clinically, complete remission is defined by a Hamilton Depression Rating Scale-17 (HAMD-17) score ≤ 7, indicating the absence or minimal presence of depressive symptoms^8,9^. MDD impairs various functional areas, including daily activities, interpersonal relationships, and work abilities. Patients who achieve remission can expect to recover pre-depression functions. Patients who achieve remission are less likely to relapse and have better psychological and occupational functioning than those in non-remission. However, even with significant improvement in depressive symptoms, some patients may still experience persistent functional impairment^10,11^. A study examining cognitive impairment in remitted and non-remitted patients with MDD^12^ discovered that patients who achieved remission after antidepressant treatment showed no significant differences from controls in overall cognitive function. However, 24% of these remitted patients exhibited at least one type of cognitive impairment, particularly in executive function and attention.

Residual mental and physical symptoms, including lack of energy, sleep disorders, and cognitive deficits, are associated with functional impairments ^13,14^. Monitoring normal functional recovery in remission is crucial, as residual functional impairments in remitted patients increase the risk of relapse ^8^. Therefore, differentiating between remitted patients with near-normal recovery and those with residual impairments can help identify high-risk individuals. This identification enables the development of customized treatment strategies to rapidly reduce relapse risk based on individual treatment prognosis.

Therefore, this study aimed to identify and verify biomarkers that can distinguish between a near-normal recovery remission state and a remission state with residual impairment through serum proteome analysis. These biomarkers can help identify remission patients with residual impairment and those at a high risk of relapse, thereby aiding in the development of rapid and precise treatment strategies.

## Methods

### Sample collection and preparation

**Fig. 1:**
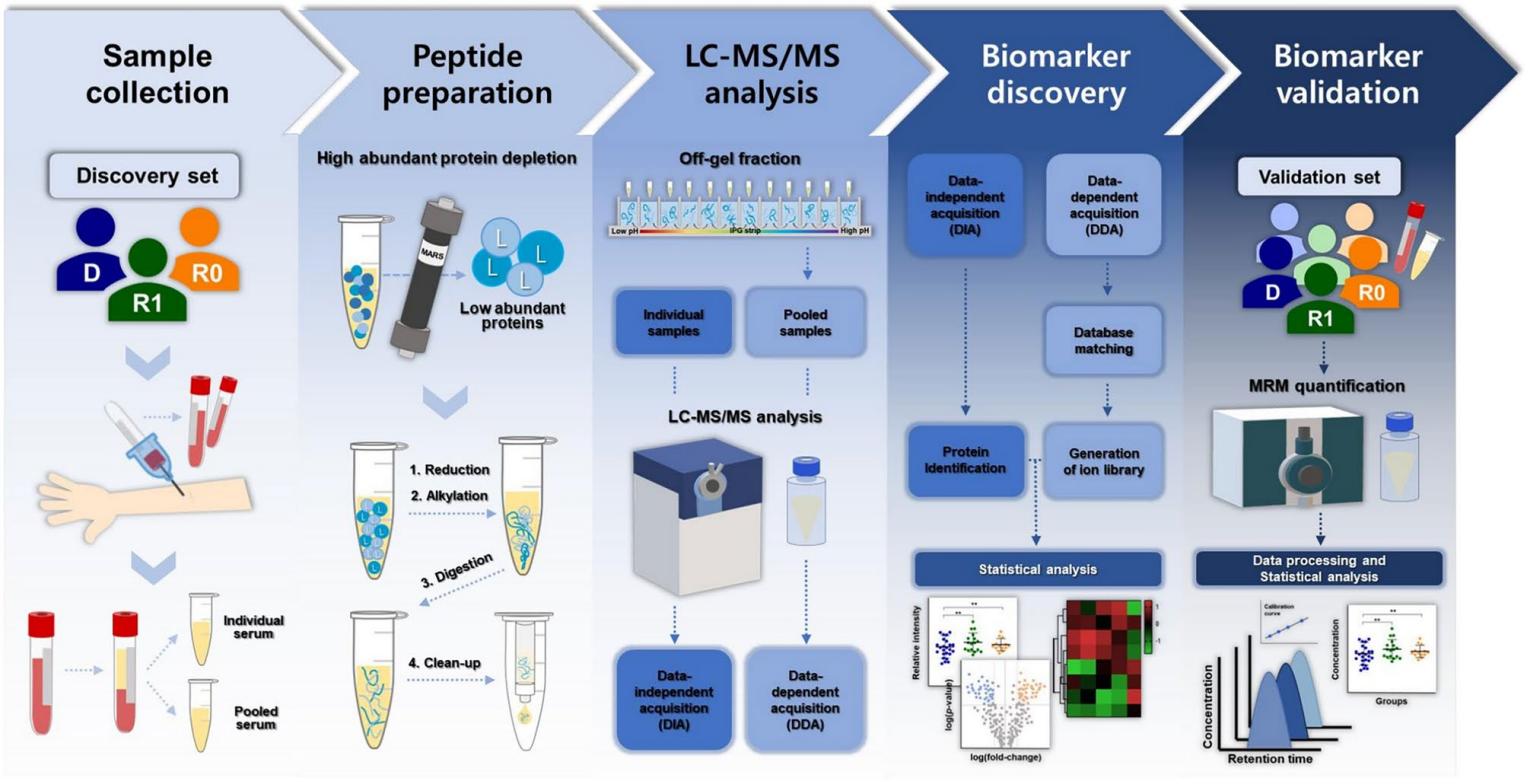
Experiment flowchart. After obtaining individual sera from experimental participants, pooled sera were prepared, and serum proteins were digested into peptides for mass spectrometry. Protein identification and biomarker discovery were performed using DDA data from pooled serum and DIA data from individual serum. MRM validation was subsequently performed on the selected marker candidates.

The Institutional Review Board of Eulji University approved this study (approval number: EU23-46, November 6, 2023), which was conducted in accordance with the latest version of the Declaration of Helsinki. Written informed consent was obtained from all participants. The study participants were a naturalistic cohort of outpatients with MDD aged 19 years or older with no history of head injury. Depression severity was assessed using the HAMD-17. Patients with depressive status were diagnosed based on a HAMD-17 score > 7. Patients in remission were classified using a HAMD-17 score of ≤ 7. Residual impairments in patients in remission referred to social, physical, and occupational functions that were less functional than before, including social relationships, work life, and learning ability, rather than sequelae such as cognitive function and sleep disturbances. These impairments were clinically diagnosed based on the comprehensive assessment of clinicians. The discovery and validation sets comprised 25 and 55 patients with MDD, respectively, and were divided into three groups: depression status (D), remission status with residual impairment (R1), and remission status with normal recovery (R0). Some patients in group D and all patients in the R1 and R0 groups were receiving medication, including antidepressants such as selective serotonin reuptake inhibitors (SSRIs), serotonin–norepinephrine reuptake inhibitors (SNRIs), and serotonin modulators, either alone or in combination. Some patients were also taking additional antipsychotic medications. Table 1 presents the patients’ demographic information. To obtain serum from each participant, blood was collected in a vacuum collection tube without added anticoagulant and centrifuged at 4000 × *g* for 5 min to separate serum from the coagulated blood. Isolated serum was stored at − 80 ℃. Samples pooled from equal amounts of serum from all participants were used to construct the ion library (Fig. 1).

**Table 1:** Demographic information.

**Patient with major depressive disorder**										
**Variables**	**Discovery set**					**Validation set**				
	**Depression ** **status** **(D)**		**Remission status ** **with residual impairment** **(R1)**	**Remission status ** **with ** **normal recovery** **(R0)**	***p*** **-value**	**Depression status** **(D)**		**Remission status with residual impairment ** **(R1)**	**Remission status ** **with ** **normal recovery ** **(R0)**	***p*** **-value**
Number of participants	10		9	6		25		17	13	
Age (mean ± SD^a^)	61.9 ± 14.3		63.1 ± 12.3	62.0 ± 16.0	0.9799^d^	47.8 ± 16.7		61.9 ± 13.0	60.8 ± 16.1	0.0083^d^
Sex (male/female)	2/8		1/8	0/6		6/19		4/13	1/12	
HAMD-17^b^ (mean ± SD)	21.7 ± 5.4		3.2 ± 2.9	3.2 ± 1.9		20.2 ± 4.3		3.5 ± 2.7	2.3 ± 1.7	
BMI^c^ (mean ± SD)	25.9 ± 3.3		23.8 ± 2.2	25.5 ± 5.6	0.4521^d^	23.6 ± 3.1		24.1 ± 2.4	24.3 ± 4.0	0.7385^e^

^a^SD, standard deviation; ^b^HAMD-17, Hamilton depression rating scale (17-item); ^c^BMI, body mass index; ^d^analysis of variance (ANOVA) with Tukey’s multiple comparisons test (D vs. R1: *p* = 0.0155; D vs. R0: *p* = 0.0452 in validation set); ^e^Kruskal-Wallis test with Dunn’s multiple comparisons test.

To deplete highly abundant proteins, a sample mixed with serum and multiple-affinity removal system buffer A at a ratio of 1:3 was injected into a multiple-affinity removal system liquid chromatography (LC) column-human 6 (human 6-HC, 4.6 × 50 mm; Agilent Technologies, Santa Clara, CA, USA). The low-abundance protein fraction was concentrated using a Nanosep Centrifugal Device with an Omega Membrane 3 K (Pall Corporation, Port Washington, NY, USA) and subsequently vacuum dried using a vacuum concentrator (Scan Vac, LaboGene, Lynge, Denmark). The vacuum-dried samples were re-suspended in buffer, and the protein concentration of each sample was determined using a bicinchoninic acid assay (Thermo Fisher Scientific, Cleveland, OH, USA). Each sample and the pooled sample contained 100 μg and 1 mg of serum protein, respectively. For reduction, samples were treated with 5 mM Tris (2-carboxyethyl) phosphine (Pierce, Rockford, IL, USA) at 37 ℃ and 400 rpm for 30 min. Subsequently, 15 mM iodoacetamide (Sigma-Aldrich, St. Louis, MO, USA) was added for alkylation, and the mixture was incubated at 400 rpm at 25 ℃ for 1 h in the dark. For digestion into peptides, mass spectrometry (MS)-grade trypsin (Promega, Madison, WI, USA) was treated at 37 ℃ and 800 rpm for 15 h. Subsequently, cleaning and desalting were conducted using a C18 cartridge (Waters, Milford, MA, USA). The digested pooled sample was fractionated into 12 fractions using an OFFGEL Fractionator (Agilent Technologies) and Immobiline DryStrip (pH 3–10; GE Healthcare, Madison, WI, USA).

## LC–MS/MS analysis for proteomic profiling

Each sample and 12 fractions of pooling samples were analyzed using the Nano-LC system Ekspert nLC415 (Eksigent Technologies, Dublin, CA, USA) coupled to the AB SCIEX 5600 triple TOF mass spectrometer (AB SCIEX, Concord, Canada). Each sample was scanned using the sequential window acquisition of all theoretical mass spectra acquisition, which is based on the data-independent acquisition method, and 12 fractions of the pooled sample were scanned using the data-dependent acquisition method. Mobile phases A and B were 0.1% formic acid in water and acetonitrile, respectively. The NanoLC trap column (0.5 mm × 350 μm; 3 μm; Eksigent Technologies) and Chrom XP NanoLC column (150 mm × 75 μm; 3 μm; Eksigent Technologies) were used. The data were analyzed in the positive ion mode, and the flow rate was 300 nL/min. The gradient program employed included the following steps: the mobile phase solution B transitioned from 5 to 40% over the initial 105 min, shifted from 40 to 90% in just 0.5 min, remained at 90% for 6 min, rapidly decreased from 90 to 5% in 0.5 min, and finally maintained at 5% for the last 8 min.

## Quantification by multiple reaction monitoring (MRM)

The inclusion criteria of the peptides used for quantification were as follows: peptides without (1) a mis-cleaved site, (2) modifications, and (3) M amino acids due to oxidation; and (4) peptides with 7–15 sequence lengths. Synthetic peptides for the selected peptide sequence were purchased (Peptron Inc., Daejeon, Korea). The transitions of peptides for quantification were extracted using Skyline software (https://proteome.gs.washington.edu/software/skyline.). The declustering potential and collision energy were optimized for each peptide transition, and the entrance potential and collision exit potential were set to fixed values of 10 and 12, respectively. MRM was performed using Sciex Exion LC and a QTRAP 5500 mass spectrometer (AB Sciex). The sample injection volume was 5 μL. An ACQUITY UPLC BEH C18 VanGuard pre-column (130 Å, 1.7 μm, 2.1 mm × 5 mm, Waters) combined with an ACQUITY UPLC BEH C18 column (130 Å, 1.7 μm, 2.1 mm × 150 mm, Waters) was used as an analytical column. Mobile phases A and B were the same as those in Sect. 2.2. The flow rate was 250 μL/min, and the final chromatogram was generated for 30 min with the following gradient: 1 min, 5% B; 20 min, 40% B; 25 min, 90% B; and 30 min, 5% B. The source parameters of the acquisition method included curtain gas = 30 psi, low collision gas, ion spray = 5500 V, 400℃, ion source gas 1 = 40 psi, and ion source 2 = 60 psi.

## Data processing and statistical analysis

The raw data from 12 fraction profiling pooled samples were imported into ProteinPilot (AB SCIEX) for protein identification and ion library construction. Subsequently, library matching was performed with the raw data of each sample using PeakView (v.2.2, AB SCIEX), and MarkerView (v.1.3.1, AB SCIEX) was used for data normalization and t-test analysis. For the *t*-test conducted to explore proteins with differences between groups, the criteria of *p* < 0.05 and fold-change ≥ 1.2 were applied. GraphPad Prism software (v.8.4.2, La Jolla, CA, USA) was used for outlier confirmation, data visualization, normality testing, difference testing, and receiver operating characteristic (ROC) curve analysis. The Shapiro–Wilk test was used to assess the normality of the data, and parametric or nonparametric statistics were selected accordingly based on the normality results. To compare the quantitative results of MRM for biomarker validation, analysis of variance (ANOVA) with post-hoc tests (Tukey’s and Dunn’s multiple comparisons tests) was used. Additionally, multiple logistic regression was applied for ROC analysis.

## Results

### Discovery of differentially expressed proteins between depression, remission with residual impairment, and near-normal remission

**Fig. 2:**
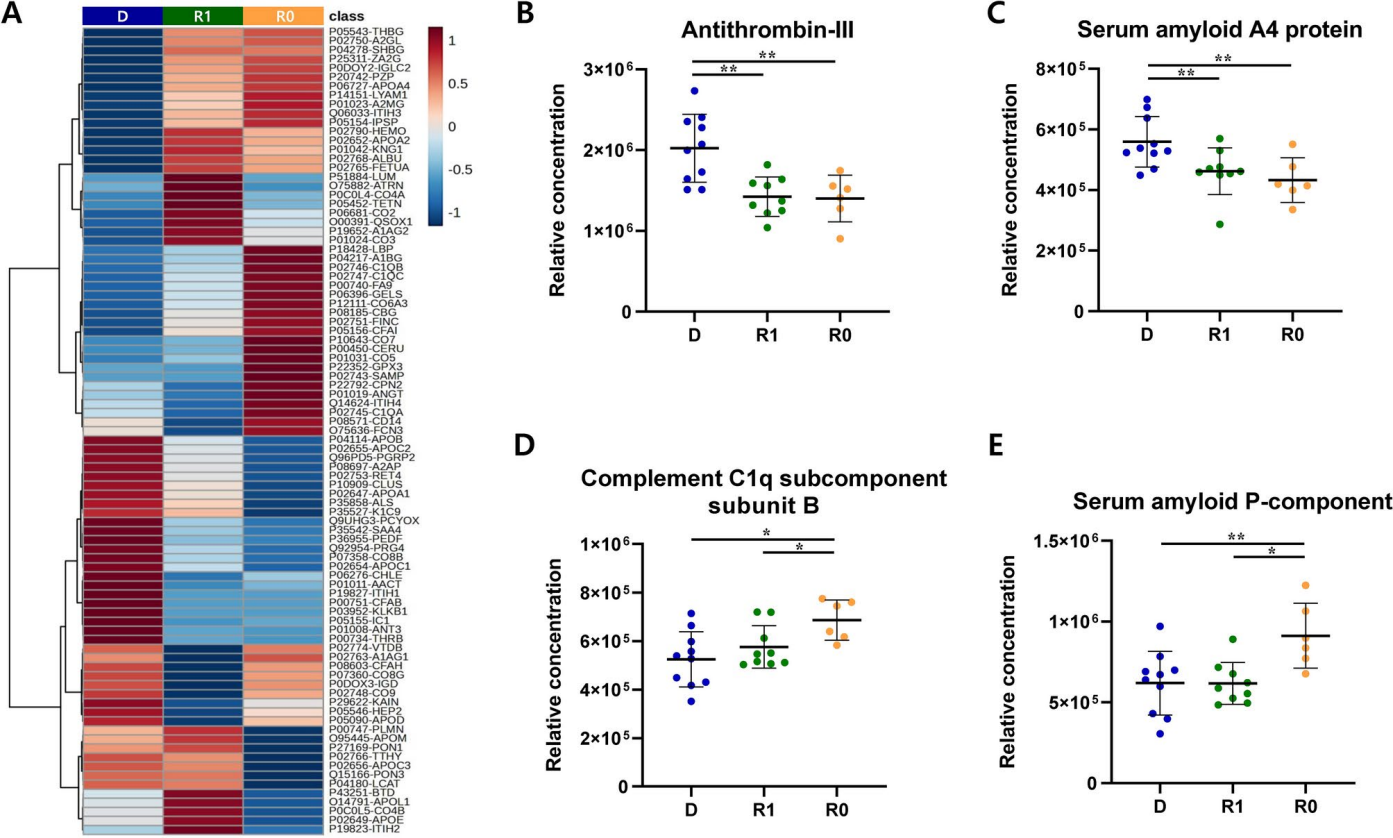
Differentially expressed proteins (DEPs) between groups. (**A**) Heatmap showing the average expression levels of the identified proteins in the depression status (D), remission status with residual impairment (R1), and remission status with normal recovery (R0) groups. DEPs had a p-value < 0.05 and fold-change < 1.2. (**B**) Antithrombin-III and (**C**) serum amyloid A4 protein are proteins that belong to the intersection between D vs. R0 and D vs. R1. (**D**) Complement C1q subcomponent subunit B and (**E**) serum amyloid P-component are proteins that belong to the intersection between D vs. R0 and R0 vs. R1. The scatter plot represents mean ± SD. *p < 0.05, **p < 0.01.

We compared serum protein profiles among the D, R1, and R0 groups to discover biomarkers that can distinguish between remission states close to normal recovery and those with residual impairment. Eighty-nine proteins were identified through ion library matching, and the group average expression levels were confirmed as a heat map (Fig. 2A). Through *t*-tests between groups, the differentially expressed proteins (DEPs) that had a *p*-value < 0.05 and fold-change ≥ 1.2 were as follows: four for D vs. R1, ten for D vs. R0, and three for R0 vs. R1. No protein was common across all three comparative combinations, but intersections were confirmed between D vs. R1 and D vs. R0 and between D vs. R0 and R0 vs. R1. Among DEPs, antithrombin-III (ANT3) and serum amyloid A4 protein (SAA4) correspond to the intersection of D vs. R1 and D vs. R0. These two proteins showed a significant decreasing trend in the remission state (whether impairment remained) group compared with the MDD group (Figs. 2B–C). The complement C1q subcomponent subunit B (C1QB) and serum amyloid P-component (SAP) correspond to the intersection of D vs. R0 and R0 vs. R1 (Figs. 2D–E). These proteins exhibited a significant increase in the remission state, which is closer to normal recovery, compared with the depressive state and remission state with residual impairment. The combination of these four proteins was selected as a candidate biomarker because it can distinguish between depressive and remission states, as well as between the complete remission state, which is closer to normal, and the remission state with residual impairment (Table 2).

**Table 2:** Differentially expressed proteins in discovery process.

No	Uniprot ID	Gene name	Protein name	D vs. R1		D vs. R0		R0 vs. R1	
				***p*** **-value**	**Fold-change**	***p*** **-value**	**Fold-change**	***p*** **-value**	**Fold-change**
1	P01008	SERPINC1	Antithrombin-III	0.0019	1.4	0.0037	1.44	0.7815	0.97
2	P35542	SAA4	Serum amyloid A4 protein	0.0095	1.22	0.0083	1.29	0.551	0.95
3	P02746	C1QB	Complement C1q subcomponent subunit B	0.3145	0.92	0.0059	0.76	0.0217	1.2
4	P02743	APCS	Serum amyloid P-component	0.8247	0.97	0.0165	0.68	0.018	1.43

## Validation of biomarker candidates and ROC analysis

**Fig. 3:**
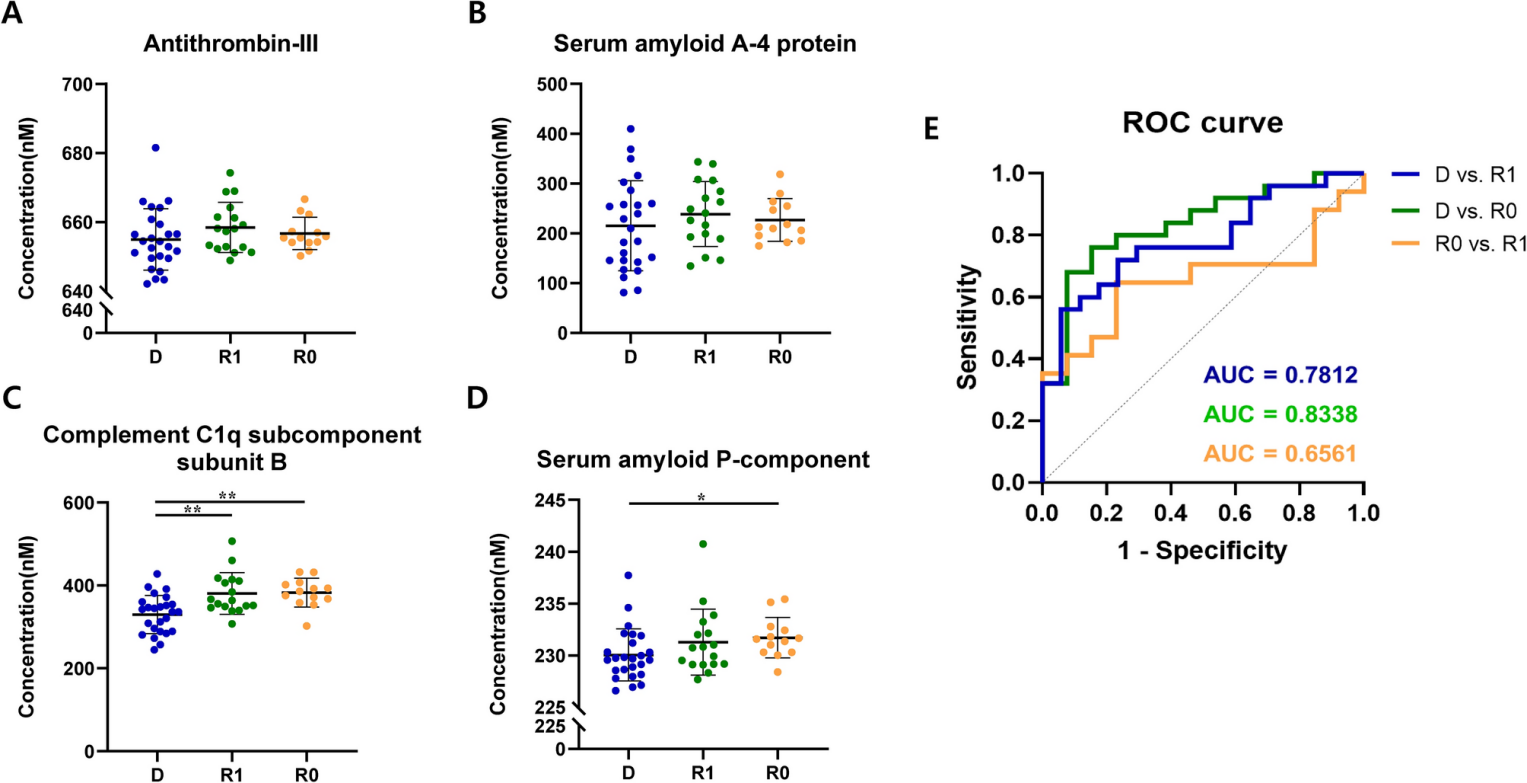
MRM quantification of protein candidates and discrimination ability evaluation. (**A**–**D**) Scatter plot showing MRM quantification of four protein candidates: antithrombin-III, serum amyloid A4 protein, complement C 1q subcomponent subunit B (C1QB), and serum amyloid P-component. The scatter plot represents mean ± SD. *p < 0.05, **p < 0.01. (**E**) Discrimination performance evaluated by the combination of C1QB and serum amyloid P-component.

MRM validation was performed for four candidate biomarker proteins, and Supplementary Table 1 presents the transitions for each peptide sequence. Figures 3A–D show the quantitative results of four proteins. No significant differences in ANT3 and SAA4 proteins were observed between groups. C1QB and SAP tended to increase in concentration, being lowest in the D group, followed by the R1 and R0 groups, and the order of increase in C1QB concentration was consistent with the discovery results. Additionally, both proteins showed significant differences between the D and R0 groups (C1QB: *p* = 0.002, SAP: *p* = 0.047). For C1QB, a significant difference was also observed between the D and R1 groups (*p* = 0.003). Furthermore, the discrimination ability of the combination of C1QB and SAP among the three groups was evaluated using ROC analysis (Fig. 3E and Supplementary Table 2). The area under the curve demonstrated the best discrimination ability for D vs. R0 to 0.8338, followed by D vs. R1 to 0.7812 and R0 vs. R1 to 0.6561.

## Discussion

This study aimed to identify and validate biomarkers that can distinguish between remission states close to normal recovery and those with residual impairments through serum protein analysis. Therefore, we confirmed DEPs in the comparison between the D, R1, and R0 groups, as well as common DEP between-group comparisons. We discovered that the combination of ANT3, SAA4, C1QB, and SAP can contribute to the distinction between disease and remission states and between remission with residual impairment and remission state close to normal. MRM validation revealed that C1QB and SAP proteins showed significant differences between patients with depression and patients who achieved normal remission. The R0 group had the highest levels, followed by the R1 and D groups, respectively. This pattern is consistent with the discovery results, indicating the potential of C1QB and SAP as biomarkers for distinguishing between a remission state close to normal recovery and those with residual impairments. If further validation confirms their effectiveness based on statistical significance, these protein markers will be useful for identifying remission patients with residual impairments at high risk of relapse and for developing faster, more precise treatment strategies.

Previous studies have reported that the four identified proteins are associated with depression. ANT3 is a glycoprotein synthesized in the liver and acts as a major natural anticoagulant by inhibiting the coagulation cascade^15^. Several studies have reported an increase in plasma ANT3 in MDD^16,17^. Following occipital repetitive transcranial magnetic stimulation (rTMS) therapy in patients with MDD, blood ANT3 levels significantly decreased, demonstrating good predictive performance for treatment response after 5 days of rTMS and a positive correlation with severity as assessed using HAMD^18^. Observations of hypercoagulability in patients with depression and reports of chronic stressors inducing low-grade coagulation suggest that ANT3-related anti-inflammatory and anticoagulant processes may influence the pathophysiology of depression^17^. Serum amyloid A (SAA) is a family of apolipoproteins related to high-density lipoproteins and is mainly synthesized in the liver^19^. SAA4 is another subtype of SAA, and SAA subtypes are expressed at different levels or in response to inflammation^19^. SAA is a multifunctional acute-phase protein that is induced by interleukin-1β and interleukin-6 and is involved in cholesterol transport and metabolism; it regulates inflammatory responses through anti-inflammatory and pro-inflammatory activities^20^. Serum amyloid A1 (SAA1), a subtype of SAA, has shown increased levels in the pooled serum of the depression group compared with the control group^21^. A cross-sectional study of inflammatory markers in psychiatric outpatients showed that higher HAMD-17 scores were significantly associated with increased SAA levels^22^. Another study observed elevated SAA in the plasma of remitted, untreated women with MDD, implying that inflammatory conditions may persist, even in clinical remission^23^. These findings suggest that persistent low-grade inflammation, even in clinical remission, may hinder the recovery of damaged functions and contribute to residual impairment. ANT3 and SAA4 did not show significant results in the validation; however, their potential as depression markers is supported by existing evidence, highlighting the need for further validation.

In this study, C1QB and SAMP proteins, which show potential for distinguishing between depressive and remission states, as well as between remission with residual impairment and near-normal remission, are also related to inflammation and immune response. C1QB is a subunit that, along with C1QA and C1QC, constitutes C1q, a component of C1^24^. C1QB is a marker of drug treatment efficacy for MDD, showing the highest levels in the control group and decreasing significantly in patients with depression treated with drugs and untreated patients, respectively^25^. C1q is an innate immune pattern recognition receptor protein that activates the classical complement pathway and plays a crucial role in preventing autoimmune diseases through complement opsonization and maintaining normal tissue homeostasis^26^. Furthermore, C1q can bind to receptors on macrophages, regulating their phenotype and inflammatory responses by, for example, inducing cytokine production and removing oxidized low-density lipoproteins^26,27^. C1q deficiency is associated with autoimmune diseases and problems with overall complement system activation are associated with inflammation and inflammation-related diseases^26^. Elevation of C1q in the central nervous system is an early response to damage. The interaction between C1q and microglial cells can effectively suppress neuroinflammation by reducing pro-inflammatory cytokine secretion, indicating that complement system-mediated neuroinflammation may involve C1q mediation^28^. In addition to the finding that C1QB, a subunit of C1q, increases as patients approach normal recovery, a previous study^29^ has shown that C1q knockout mice exhibited electric foot shock-induced learned helplessness behavior, with significantly reduced C1q messenger ribonucleic acid levels in the frontal cortex. These results suggest that complement pathway components and their activity may influence the pathophysiological mechanisms of depression associated with inflammation. Furthermore, the production of C1 complexes in neurons in the central nervous system in response to inflammatory pathways and the activation of microglia and astrocytes through C3 can increase neurotoxicity, leading to neuronal damage and death, which may contribute to synaptic loss and cognitive impairment^28^. Inflammation-related structural and functional changes can occur in the hippocampus and prefrontal cortex, with chronic immune dysregulation or inflammation and excessive release of pro-inflammatory cytokines being key pathological features of relapsed MDD^30^. Therefore, residual social, occupational, physical, and functional impairments in remitted depression may be related to extensive neurological system damage due to persistent inflammation. These pathophysiological effects could be associated with a higher risk of depression relapse.

SAP, also known as pentraxin-2, is a glycoprotein belonging to the pentraxin protein family and is synthesized and secreted by hepatocytes under normal conditions^31^. As a pattern recognition protein, SAP is involved in innate immunity, similar to C-reactive protein and pentraxin-3, and can activate the classical complement pathway through interaction with C1q^32^. SAP is also a ubiquitous component of human amyloid deposits, contributing to the persistence and formation of amyloid, and is associated with Alzheimer’s disease^33^. A study comparing plasma levels of SAP at baseline, 2 weeks, and 12 weeks in patients with MDD receiving escitalopram monotherapy (an SSRI treatment) for 8 to 12 weeks^34^ discovered a significant increase in SAP levels in the depression group compared with the control group at baseline. Additionally, SAP levels significantly decreased over the course of treatment. The relationship between changes in SAP levels and the alleviation of depression symptoms remains unclear. The proposed biological mechanism for increased SAP in depression is as follows: 1) Aβ increases through direct interaction with Aβ peptide at the carbohydrate-binding site of SAP; 2) central SAP, Aβ, and complement C1q attract and activate microglia; and 3) peripheral SAP regulates macrophage activation through Fcγ receptors^34^. This mechanism may enhance the release of inflammatory cytokines, promoting macrophage activation and neuronal cell death^34^. In this study, SAP levels tended to increase as remission approached normal; this is potentially related to inflammation. Decreased SAP in the depressed state may indicate abnormal complement activity and immune processes involving C1q. Symptom remission might result from immune responses such as anti-inflammatory activities, leading to increased SAP levels for further immune response activity. Furthermore, the neurobiological mechanisms that preserve cognitive function in MDD remain to be fully elucidated. However, the degree and severity of cognitive dysfunction are mediated by the location of inflammation, neurotoxicity, and apoptosis^11^. This finding suggests that residual social, physical, and occupational impairment in remitted depressed patients may result from cognitive dysfunction associated with immune and inflammatory response mechanisms.

**Fig. 4:**
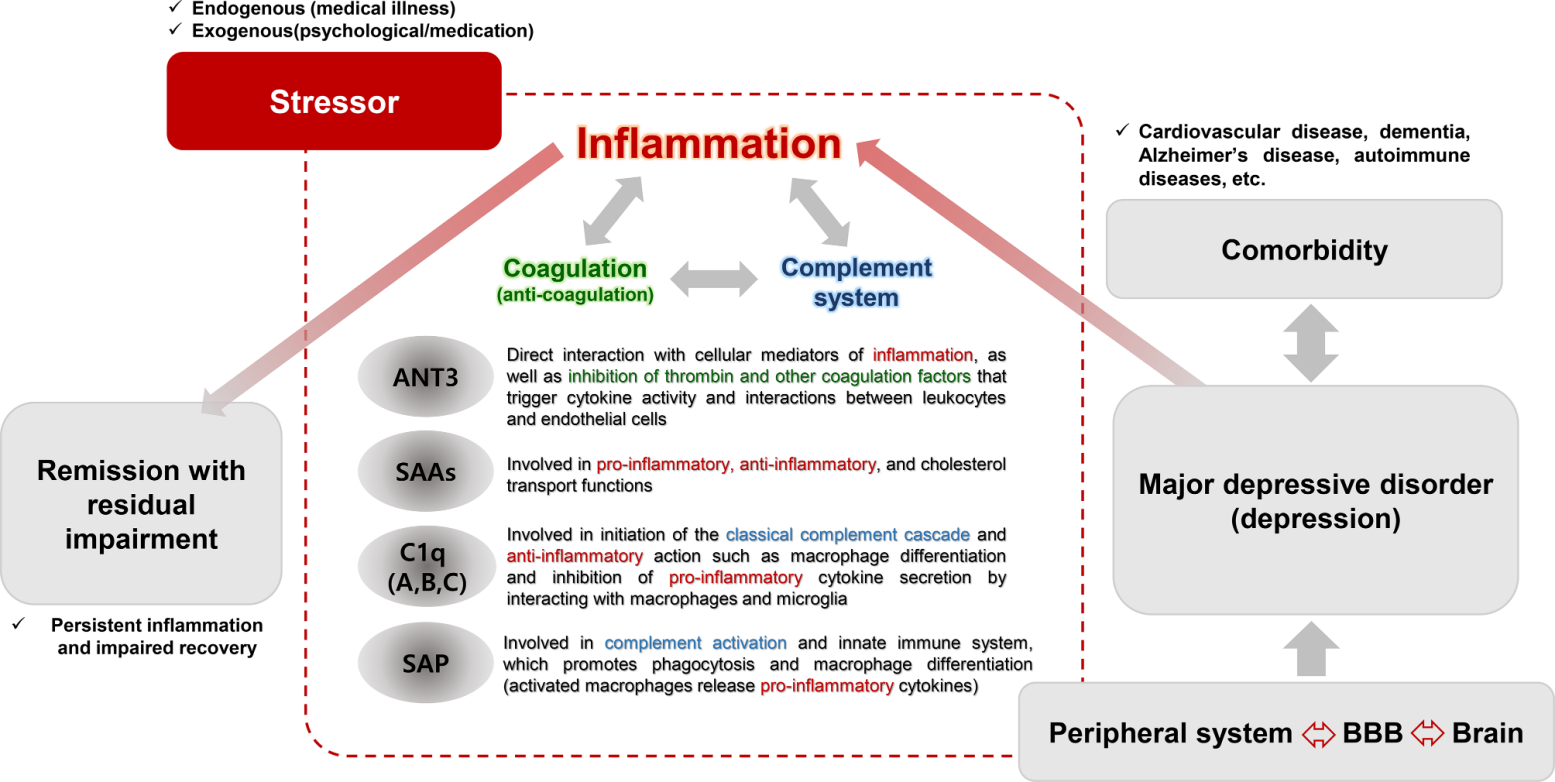
Putative schematic diagram of residual impairment in major depressive disorder remission. Inflammation can be activated in response to stressors. The four proteins discovered in this study––antithrombin-III (ANT3), serum amyloid A4 protein (SAA4), C1QB, and serum amyloid P-component––are related to inflammation, complement system, and anticoagulation, and these pathways interact with each other. Potentially, inflammation may contribute to the pathogenesis of depression, comprehensively affecting the peripheral-blood–brain barrier (BBB)-brain zone. The periphery and brain communicate bidirectionally. Inflammation that persists even in remission of major depressive disorder can impede recovery, resulting in residual symptoms (such as cognitive dysfunction) and, consequently, occupational, physical, and social impairments. Changes in inflammation, the complement system, and coagulation pathways may be associated with depression and bidirectional comorbidities.

The four proteins discovered in this study are related to the complement system, inflammation, and coagulation processes. The pathophysiology of inflammation activated by various stress factors is closely linked to depression^35–37^. Among the immune processes associated with inflammation, evidence of altered levels of immune-related proteins suggests that residual social, occupational, and physical dysfunction in remitted MDD may stem from cognitive and functional impairments driven by inflammatory and immune response mechanisms (Fig. 4). The interaction between the complement and coagulation systems can contribute to various diseases^38^. Targeted research on these interactions can help explore the relationships and molecular mechanisms between depression and comorbidities that have a bidirectional relationship with depression. SAA is a well-known indicator of cardiovascular disease and interacts with high-density lipoprotein function to alter the biological effects under certain clinical conditions^39^. Additionally, studies have discovered a higher prevalence of MDD in patients with hypercholesterolemia^40^ and higher levels of total cholesterol, apolipoprotein B, and high-density lipoprotein-cholesterol in the MDD group compared with the control group^41^. These findings highlight the complex and bidirectional association between depression and cardiovascular disease. As inflammatory cells, platelets can express several complement receptors, and the binding of these receptors to C1q causes platelet activation and coagulation^38^. SAP is involved in C1q-mediated complement pathway activity and plays an important role in cardiovascular processes such as fibrosis, coagulation, and inflammation^31^. ANT3 has anticoagulant and inflammatory response control functions, and the observation of high procoagulant index and fibrinogen expression in patients with MDD in remission suggests a relationship between MDD, improved coagulation promotion, and cardiovascular disease risk^18^.

This preliminary study identified potential biomarker candidates for screening patients with residual impairments in depression; however, there are limitations in the interpretation and discussion. The study participants consisted of a small number of psychiatric outpatients. As a naturalistic cohort with less strict control over medication use and lifestyle, the study offers the advantage of producing results that are closer to clinical applicability. Patients used antidepressants either alone or in combination with other classes of antidepressants, and some were prescribed additional antipsychotics. Various medication-related factors may affect the identified immune and inflammatory pathways. In addition to medication variables, age and the presence of comorbidities should be considered in studies involving a depressed population, as the prevalence of depression increases with age. Age differences between study participant groups may indicate different bioenvironments. In this study, group D in the validation set included more younger patients than groups R1 and R0, suggesting that age differences may have influenced the differences in biomarker levels in the validation data. Physical conditions such as cardiovascular disease, diabetes, hypertension, and chronic pain, which are common in older patients, may have a bidirectional relationship with depression^42^ and may share mechanisms related to the inflammatory hypothesis, one of the proposed pathophysiological mechanisms of depression. Future studies with larger sample sizes, controlled medication use (to confirm the effects of a single drug), and consideration of demographic variables (to control for confounding factors) may provide further evidence on the pathophysiological mechanisms associated with residual impairments and the treatment of depression.

## Conclusion

The trend of protein changes between depressed states, remission states with residual impairment, and near-normal remission states suggests that C1QB and SAP could serve as biomarkers for distinguishing remission with residual impairment. Inflammatory responses can trigger depression and hinder recovery. Alterations in proteins involved in coagulation (anticoagulation), the complement system, and inflammatory response provide insight into the pathophysiological mechanisms behind the persistence of impairments in patients with MDD who have achieved remission. These findings also shed light on the bidirectional comorbidity between depression and cardiovascular disease.

## Data availability

The data that support the findings of this study are available from the corresponding author upon reasonable request.

## Supplementary Information

Supplementary Information 1.

Supplementary Information 2.
