# Supplementary material for: Serum proteomic changes related to residual impairment in remittent depression are associated with immune and inflammatory processes
Source: Sci Rep. 2024 Oct 18;14:24482. doi: 10.1038/s41598-024-75983-0 (PMC11489708; doi:10.1038/s41598-024-75983-0)
Supplement: Supplementary file 1 — Supplementary Information 1. [file 41598_2024_75983_MOESM1_ESM.docx]

**Supplementary table 1. MRM parameters of protein candidates**

| **No.** | **Uniprot ID** | **Protein name** | **Peptide sequence** | **Q1 (mass, Da)^*^** | **Q3 (mass, Da)** | **Q3 ion type** | **DP** | **CE** |
| --- | --- | --- | --- | --- | --- | --- | --- | --- |
| 1 | P01008 | Antithrombin-III | TSDQIHFFFAK | 670.835 | 147.113 | 2y1 | 60 | 39 |
|  |  |  |  | **670.835** | **432.173** | **2b4** | **60** | **37** |
|  |  |  |  | 670.835 | 576.795 | 2y9+2 | 60 | 33 |
| 2 | P35542 | Serum amyloid A4 protein | EALQGVGDMGR | **566.774** | **691.319** | **2y7** | **80** | **29** |
|  |  |  |  | 566.774 | 535.229 | 2y5 | 80 | 25 |
|  |  |  |  | 566.774 | 363.181 | 2y3 | 80 | 39 |
| 3 | P02746 | Complement C1q subcomponent subunit B | LEQGENVFLQATDK | 796.402 | 1221.581 | 2y11 | 89.2 | 34.5 |
|  |  |  |  | 796.402 | 822.426 | 2y7 | 89.2 | 36.5 |
|  |  |  |  | **796.402** | **675.317** | **2y6** | **89.2** | **32.5** |
| 4 | P02743 | Serum amyloid P-component | AYSLFSYNTQGR | **703.839** | **972.453** | **2y8** | **82.4** | **34.2** |
|  |  |  |  | 703.839 | 825.385 | 2y7 | 82.4 | 34.2 |
|  |  |  |  | 703.839 | 235.108 | 2b2 | 82.4 | 34.2 |

*Transition used for quantification
