# Supplementary material for: Serum proteomic changes related to residual impairment in remittent depression are associated with immune and inflammatory processes
Source: Sci Rep. 2024 Oct 18;14:24482. doi: 10.1038/s41598-024-75983-0 (PMC11489708; doi:10.1038/s41598-024-75983-0)
Supplement: Supplementary file 2 — Supplementary Information 2. [file 41598_2024_75983_MOESM2_ESM.docx]

**Supplementary table 2. confusion matrix of ROC**

| **Observed** | **Predicted** | | **Total** | **% Correctly classified** |
| --- | --- | --- | --- | --- |
|  | **R1** | **D** |  |  |
| **D** | 5 | 20 | 25 | 80.00 |
| **R1** | 7 | 10 | 17 | 41.18 |
|  | **R0** | **D** |  |  |
| **D** | 4 | 21 | 25 | 84.00 |
| **R0** | 7 | 6 | 13 | 53.85 |
|  | **R0** | **R1** |  |  |
| **R1** | 1 | 16 | 17 | 94.12 |
| **R0** | 1 | 12 | 13 | 7.69 |

D, depression status; R1, remission status with residual impairment; R0, remission status with normal recovery.
